# Assessing inequalities in publicly funded health insurance scheme coverage and out-of-pocket expenditure for hospitalization: findings from a household survey in Kerala

**DOI:** 10.1186/s12939-023-02005-2

**Published:** 2023-09-27

**Authors:** Santosh Kumar Sharma, Jaison Joseph, Hari Sankar D, Devaki Nambiar

**Affiliations:** 1 The George Institute for Global Health, New Delhi, India; 2 Faculty of Medicine, University of New South Wales, Sydney, Australia; 3 Prasanna School of Public Health, Manipal Academy of Higher Education, Manipal, India

**Keywords:** Publicly funded health insurance, Universal health coverage, Out-of-pocket expenditure, Schemes for health

## Abstract

**Background:**

Increasing financial risk protection is a key feature of Universal Health Coverage and the path towards health for all. Publicly Funded Health Insurance Schemes (PFHIS) have been considered as one of the pathways to safeguard against financial shocks and potentially reduce Out-of-Pocket Expenditure (OOPE). The south Indian state of Kerala has roughly a decade-long experience in implementing PFHIS. To date, there have been very few assessments of the coverage of these schemes and their impact on expenditure. Aiming to fill this gap, we explored the extent of and inequalities in insurance coverage, as well as choice of providers, and median cost of hospitalization in Kerala among insured and uninsured individuals.

**Methods:**

A cross-sectional household survey was conducted in four districts of Kerala as part of a larger health systems research study from July–October 2019. We employed multistage random sampling to collect data from 13,064 individuals covering 3234 households in the catchment area of eight primary health care facilities. We used descriptive statistics, bivariate and multivariate analysis. We evaluated socioeconomic disparities using an absolute measure of inequality—the Slope Index of Inequality (SII) and a relative measure—the Relative Concentration Index (RCI).

**Results:**

A substantial proportion of our study respondents reported that they were covered by PFHIS (45.8%). Respondents belonging to lowest and middle wealth quintiles of household had significantly greater odds of being covered by insurance than respondents belonging to the richest wealth quintile. The negative magnitude of RCI [-16.8% (95%CI: -25.3, -8.4)] and SII [-21.5% (95%CI: -36.1, -7.0)] suggest a higher concentration of PFHIS coverage among the poor. Median OOPE for hospitalisation at private health facilities was INR 9000 (approx. USD 108.70) among those covered by PFHIS, whereas it was INR 10500 (approx. USD 126.82) at private health facilities among those not covered by insurance.

**Conclusion:**

While PFHIS seems to be appropriately targeting poorer populations, among the insured, OOPE for hospitalization persists. Among the uninsured, population subgroups with advantage are spending the greatest amount, raising questions about whether those facing relative disadvantage are forgoing care altogether or seeking care using cheaper, public avenues. Further policy action to more effectively reduce financial burden among left behind eligible populations under PFHIS will be essential to UHC progress in the state.

## Background

Sustainable Development Goal (SDGs) 3.8 of the United Nations calls for countries to progressively achieve Universal Health Coverage (UHC) through coverage of a wide range of services across population subgroups, assuring financial risk protection [1, 2]. For this, latter commitment of increasing financial risk protection and averting catastrophic expenditure, Publicly Funded Health Insurance Schemes (PFHIS) are considered a key strategy in many developing countries [3, 4]. PFHIS in developing countries may improve service access and avert financial catastrophe among those seeking in-patient care [4–6]. There remain many gaps, however.

A wide range of PFHIS programs were introduced by various state governments as well as the national government over the past two decades. The Universal Health Insurance scheme was introduced by the Central government in 2003 and later revamped in 2008 to become the Rashtriya Swasthya Bhima Yojana (RSBY) [7]. This scheme offered health insurance coverage of Rs.30000/- (approx. USD 364.88) to five members of Below Poverty Line (BPL)^1^ families for hospitalization on a floater basis^2^ [7, 8].

Many studies have evaluated the effects of PFHIS in India with regards to enrolment, utilization, Out-of-Pocket Expenditure (OOPE), and access to healthcare [9–16]. A majority of the studies report that while PFHIS have had little to no impact in reducing OOPE [9, 10, 13, 15, 17], they have improved access and utilization of healthcare services [14–16]. The majority of the data on the impact of PFHIS in India shows that it has not succeeded in providing financial security [4, 5, 17–23]. A few studies have documented reduced out-of-pocket expenditure (OOPE) as a result of PFHIS [24–26]. According to some studies, there has been an increase in inpatient care usage as a result of PFHIS [17, 18, 21, 24]. On the contrary some studies have not found evidence of increased hospital utilization due to PFHIS [4, 22].

Kerala, a state in South India, has prioritized welfare schemes and gained over a decade of experience in implementing PFHIS. Kerala’s journey began with pilots of the RSBY scheme in Kollam and Alappuzha districts in 2008 [27]. The scheme was scaled up to all 14 districts of the state in the same year with further addition of eligible beneficiaries under the scheme [27]. Families belonging to the BPL category listed by the Central government were provided coverage under the RSBY scheme, while families listed by the state were covered under the Comprehensive Health Insurance Scheme (CHIS) [27]. The option for Above Poverty Line (APL) families in the state to get covered under this health insurance scheme was launched during the initial years of implementation of CHIS, where these families had to pay a premium to enjoy the benefits of the scheme [28], this facility was discontinued in later years. In 2011, the Kerala government introduced an additional support amount of Rs.70000/- (∼USD 850) to RSBY and CHIS eligible families seeking care for chronic disease conditions relating to heart, kidney, liver, and trauma care [29].^3^

In 2017, the state implemented the Senior Citizens Comprehensive Health Insurance Scheme (SCHIS) which provided an additional coverage of Rs. 30000/- (∼USD 364.88) for hospitalization of people aged above 60 years beyond [32]. Apart from the PFHIS targeting the poor and informal sector in the state, Kerala also implements the Employees State Insurance Scheme (ESIS) targeting workers in the formal sector and the Central Government Health Scheme (CGHS), which caters to civil servants/ those in government service [33].

Despite the state having a decade-long experience in implementing PFHIS, the burden of OOPE in the state remains large [34–36]. The recent National Health Accounts (NHA 2018–19) indicate that Kerala has the highest OOPE on health at Rs. 6,772 per capita (∼USD 82) at the point of receiving health care by households when compared with other states in the country [37]. National Sample Survey (NSO) 75th round also found higher out-of-pocket medical expenses (Rs.4,469, USD 54) and in the private sector hospitals (Rs.28,775, USD 350) in Kerala when compared with other Indian states [38]. This suggests that Kerala follows the national trend of PFHIS increasing service utilisation and not reducing OOPE.

As part of a larger health systems study, we carried out an analysis of primary household survey data to determine how PFHIS coverage relates to service utilisation and expenditure comparing the insured to the uninsured. The main objective of this study was to identify the factors that contribute to PFHIS coverage and socio-economic inequalities in its coverage in Kerala. We further analysed how hospitalisation and out-of-pocket expenditure on hospitalisation were distributed by PFHIS coverage across public and private health facilities, as well as socio-demographic and economic subgroups in the state.

## Materials and methods

### Study setting

A cross-sectional household survey was conducted in four districts of Kerala as part of a larger health systems research study from July–October 2019. All fourteen districts of the state were grouped into four categories based on an index generated using selected health indicators and data on determinants of health sourced from the National Family Health Survey (NFHS 2015–16). One district per group was randomly chosen, and one Family Health Centre (FHC) and one Primary Health Centre (PHC) in that district were randomly selected. Household level data collection was conducted in the institutional catchment areas following a multi-stage random sampling. Detailed Information on data collection, household survey methods and sampling size calculation is reported elsewhere [39].

### Study tools

We gathered information from 3,234 households and the questionnaire consisted of various sections covering individual demographics, health information, hospitalization, outpatient care, and out-of-pocket expenses. Information on selected indicators were collected using data from different subsamples. The indicators selected in this study are categorized into following: (i) total population covered by any Insurance (ii) insurance coverage by type of insurance, (iii) choice of inpatient care by insured and uninsured population, and (iv) mean and median out-of-pocket expenditure (OOPE) for hospitalization by insured and uninsured population.

Data on insurance coverage was obtained from 13,064 individuals by asking them whether they were covered or not covered under any type of insurance. Participants who were covered under any insurance scheme were asked to specify the scheme in which they were enrolled (PFHIS (RSBY/CHIS/AB-PMJAY/KASP), Central Government Health Insurance Schemes (CGHS,ESIS,ECHS etc.), State Government Health Insurance scheme for employees (MEDISEP), community health insurance, health insurance provided by a micro finance institution, health insurance provided by private employer, private health insurance, or others). We analysed 13,054 cases but had to exclude 10 transgender^4^ persons in our sample as we were underpowered to make any inferences about this population subgroup. We categorized the extent of health insurance coverage under three groups; population covered under PFHIS, population covered under private or community based insurance, and population not covered by any insurance. A total sample of 11,832 people (excluding 1222 cases of non-PFHIS coverage) of individuals covered under PFHIS were included for this analysis.

Information regarding inpatient care was obtained from 1055 participants who said they had experienced hospitalization at any point 365 days prior to the survey. From these individuals, we collected data on (i) nature of ailment, (ii) type of care and (iii) medical and non-medical expenditures incurred for inpatient visits. We excluded 104 individuals who were covered under non-PFHIS and 951 cases of those who were covered by PFHIS and not covered by any insurance are included for this analysis.

### Variables used in this study

#### Outcome variable

PFHIS coverage was the primary outcome variable for this study which was created using the information from response give by participants on their status of enrolment under any insurance schemes. For this analysis we excluded individuals covered by any private and other social health insurance schemes, as they were small proportion (2.74% in CGHS, ESIS, ECHS etc., 3.03% in State Government Health Insurance scheme for employees, 3.29% in community health insurance, 0.08% in health insurance provided by a micro finance institution, 0.01% in health insurance provided by private employer, and 0.22% private health insurance, or others) of our overall sample.

Therefore, we were comparing the population covered by PFHIS to those not covered by any health insurance.

Another outcome variable was Out- of -Pocket expenditure (OOPE) on hospitalization. Information on OOPE was collected as a part of hospitalization within a reference period of 365 days prior to the survey. Information was collected across nine sub-components: Service fee (includes doctors’ fees/ bed charges/ OT charges), diagnostic tests, medicines & consumables (from the hospital/clinic visited or from outside), lodging of the escort/attendant, transportation costs for patient, informal payments, and other medical expenses (attendant charges, expense for physiotherapy, personal medical devices, blood, and oxygen). The OOPE was derived from the total expenditure minus reimbursement.

#### Independent variable

Following convention, we included sex (male, female), marital status (never married, currently married, currently not married), religion (Hindu, Muslim, Others (Christian, Sikhs, Jain, and others.)), caste (Scheduled Caste [SC], Scheduled Tribe [ST], Other Backward Caste [OBC], Other),^5^ and household wealth quintile (poorest, poorer, middle, richer, richest), status of hospitalization in the past year and sector of hospitalization (no or yes and for yes, whether in public or private sector).

To determine the economic status of households, a wealth index was used. This index was established by considering various economic indicators such as household assets, access to safe water and sanitation, and land ownership. The data was separated into dichotomous variables, and principal component analysis (PCA) was applied to assign weights to each indicator. The resulting wealth index was categorized into five quintiles ranging from the poorest to the richest [42]. Hospitalization in public and private hospitals was determined by a question which asked hospitalised individuals “where did you seek care?”. Public hospitalization includes Sub centre/ASHA/AWW etc., PHC, FHC, CHC, Sub District/Taluk Hospital, District Hospital, Medical College Hospital, ESI/ECHS/ CGHS Hospital, General Hospital, Women and Child Hospital, Government supported/subsidised/Jan dhan/Karunya pharmacies, Public Ayurveda facility, Public Homeopathy facility and Other public health facility. Private hospitalization includes private doctor/ clinic, private nursing home, private hospital, charitable/ Trust Hospital, private multi/ super specialty hospital, private medical college, private pharmacy, private lab, Registered Medical Practitioner, Traditional healer, private Ayurveda doctor, private Ayurveda facility, private Homeopathic doctor, private Homeopathic facility, and other private health facilities.

### Analysis

The analysis approach used both descriptive and statistical analyses, including bivariate and multivariate analyses. Descriptive statistics displayed the distribution of participants according to independent and outcome variables. Categorical variables were expressed in frequencies and proportions, with all proportions calculated after eliminating missing data.

We utilized bivariate analysis to explore the correlation between independent variables and PFHIS coverage, the outcome variable. Furthermore, we employed multivariable logistic regression analyses to investigate the connection between independent and dependent variables. To conduct multivariate analyses, the regression model included significant variables from the bivariate analysis (p < 0.05) such as gender, marital status, hospitalization in the past year, wealth quintile, social group, and religion. This was done to control any potential confounding factors between them. The findings of the logistic regression study provided estimates of odds ratios, adjusted and unadjusted, based on sociodemographic characteristics, accompanied by 95% confidence intervals.

To access the socioeconomic inequality in PFHIS coverage in Kerala, concentration curve (CC), relative concentration index (RCI) and slope index of inequality (SII) were used. RCI was used to measure relative inequality and SII was used to measure absolute inequality [43–45]. The SII is a measure of the difference between predicted health indicator values for the richest and poorest wealth quintiles. It accounts for the entire distribution of the stratification variable using an appropriate regression model. The goal is to ensure accurate and fair assessment of the health indicator differences across socioeconomic groups [43, 45, 46]. The calculation of SII is generally based on a linear regression model. However, the logistic regression model is more suitable for its computation. This is because it is typically used to assess the coverage of indicators and the prevalence of health outcomes. Additionally, it avoids making linear predictions that fall outside the expected interval of a proportion, which ranges from 0 to 100 [43]. In terms of proportions, the absolute difference between the group and the SII falls within the range of -100 to 100 percentage points. Values of SII close to zero mean there is no inequality, negative values revealed that the indicator is concentrated in disadvantaged households, while a positive value of SII indicates that the indicator is concentrated in the most advantaged groups.

A relative summary measure is unique because it lacks units, making it easier to compare different indicators. The RCI value is equivalent to twice the area between a diagonal line representing perfect equality among groups and the curve that shows the coverage for each cumulative percentage of the population studied. If coverage is higher among the wealthiest individuals in the top quintile, the area generated is below the diagonal line. Conversely, if coverage is higher among the poorest individuals in the bottom quintile, the area generated is above the diagonal line [47]. The RCI values range from -1 to + 1, with zero representing equality. The further the values are from zero, the greater the relative inequality [43, 44, 48]. In our study, we multiplied the SII and RCI results by 100 to make them easier to visualize in tables and graphs, with a range of -100 to + 100.

Further, we analyzed the proprotions of hospitalized individuals by PFHIS coverage according to gender, marital status, hospitalization in the past one year and by wealth quintile, social group, and religion. We have also estimated mean and median OOPEfor hospitalization among those covered by PFHIS and individuals not covered by any insurance.

The statistical analyses were performed using Stata®17 MP version (StataCorp LLC, Lakeway Drive College Station, Texas, USA), with the relevant sampling weight variables applied in the dataset.

### Ethics approval

The Institutional Ethics Committee of the George Institute for Global Health granted approval for the study (Project Number 05/2019). Additional permissions were obtained from Department of Health and Family Welfare (DHFW) Kerala. While conducting the study, each health facility and concerned local self-government body was appraised about the purpose of the study. Participants provided written consent and data was stored securely on password-protected servers.

## Results

### Participant characteristics

The study participants' socio-demographic characteristics are summarized in Table 1. About 53% of our sample comprised females and 47% comprised males (in addition to this, we had a sample of 10 trans individuals whom we could not include in subsequent analyses). Around 55% respondents were currently married. More than one -third of the participants belonged to the bottom two quintiles (poorest and poorer). About more than three-fifths belonged to the OBC category (62.8%). Most of the respondents belonged to Hindu religion (65%) followed by Muslims (18.5%) and Christian or others (16.5%). It was observed that about 8.2% respondents had been hospitalized in the previous year.

**Table 1:** Sample characteristics of respondents in Kerala, 2019

Background characteristics	N	%
**Gender**		
Male	6204	47.4
Female	6850	52.6
**Marital Status**		
Never married	2777	21.4
Currently Married	7265	55.5
Currently not married	3012	23.1
**Whether hospitalised?**		
No	11976	91.8
Yes	1078	8.2
**Wealth Quintile**		
Poorest	2345	17.4
Poorer	2590	19.8
Middle	2684	20.3
Richer	2718	21.1
Richest	2717	21.5
**Social Group**		
Scheduled Caste (SC)	947	7.4
Scheduled Tribe (ST)	234	2.1
Other Backward Class (OBC)	8392	62.8
Others	3481	27.7
**Religion**		
Hindu	9185	65.0
Muslim	2186	18.5
Christian and others	1683	16.5
**PFHIS coverage**		
No insurance	5866	45.37
PFHIS	5966	45.79
Non-PFHIS	1222	8.84
**Total**	13054	100.0

Non-PFHIS include private insurance, social health insurance etc. This sample was excluded from subsequent analyses

### Association between PFHIS coverage and participants characteristics

Table 2 presents the percentage distribution of respondents by PFHIS coverage according to selected background characteristics. Table 2 also present the results of association between PFHIS coverage and background characteristics of participants. Overall, equal proportion of respondents were covered by PFHIS and not covered by any health insurance /schemes in Kerala. Bivariate analysis of PFHIS coverage by gender shows that a slightly greater proportion of females (51.3% females vs 49% males) was covered by PFHIS. Adjusted analysis also showed that females respondents had significantly higher odds [AOR: 1.17, 95% CI: 1.08, 1.26] of being covered by PFHIS than males’ counterparts. Never married respondents had significantly lower odds [AOR: 0.82, 95%CI: 0.73, 0.88] of being covered by PFHIS than the currently married respondents. A negative gradient was observed in the PFHIS coverage among respondents moving from poorest wealth quintile (57.5%) to richest wealth quintile (37%). Adjusted analysis also showed that odds of being covered by PFHIS decreased from poorest wealth quintile to richer wealth quintile, however, respondents belonging to the poorest [AOR:2.21, 95%CI: 1.94, 2.51], poorer [AOR: 2.09, 95%CI: 1.85, 2.37], middle [AOR: 2.06, 95%CI: 1.82, 2.34] and richer wealth quintile [ AOR: 1.67, 95%CI: 1.48, 1.89] of households had significantly greater odds of being covered by PFHIS than the richest wealth quintile. A greater proportion of respondents belonging to SC group (60.7%) were covered by PFHIS followed by Others (51.0%), OBC (48.8%), and ST(45.7%). Respondents belonging to SCgroup had significantly higher odds [AOR: 1.29, 95%CI: 1.10, 1.51] of being covered by PFHIS after controlling for other variables in the logistic regression analysis. Respondents belonging to ST and OBC caste group had significantly lower odds of being covered by any health insurance. PFHIS coverage was higher among respondents belonging Hindu (53.7%), followed by Muslim (44.9%) and Christian & Others religious group (43.1%). It was found that respondents belonging to Muslim [AOR: 0.76, 95%CI: 0.69, 0.84] and Christian or other religious groups (AOR: 0.65, 95%CI: 0.58, 0.73] had significantly lower odds of being covered by PFHIS. Respondents who were hospitalized in the past year had 22% higher odds [AOR:1.22, 95%CI: 1.07, 1.40] of being covered by PFHIS than those not hospitalised.

**Table 2:** PFHIS Coverage by background characteristics in Kerala

Background characteristics	N	PFHIS (%)	No insurance (%)	AOR [95% CI]
**Gender**				
Male^a^	5,603	49.1	50.9	1.00
Female	6,229	51.3	48.7	1.17*** [1.08, 1.26]
**Marital Status**				
Never married	2,522	49.6	50.4	0.82*** [0.73, 0.88]
Currently Married^a^	6,521	56.8	43.2	1.00
Currently not married	2,789	35.5	64.5	0.44*** [0.40, 0.48]
**Wealth Quintile**				
Poorest	2,290	57.5	42.5	2.21*** [1.94, 2.51]
Poorer	2,503	54.1	45.9	2.09*** [1.85, 2.37]
Middle	2,521	54.2	45.8	2.06*** [1.82, 2.34]
Richer	2,479	47.3	52.7	1.67*** [1.48, 1.89]
Richest^a^	2,039	37.0	63.0	1.00
**Social Group**				
Scheduled Caste (SC)	911	60.7	39.3	1.29*** [1.10, 1.51]
Scheduled Tribe (ST)	232	45.7	54.3	0.63** [0.48, 0.83]
Other Backward Class (OBC)	7,734	48.8	51.2	0.87** [0.80, 96]
Others^a^	2,955	51.0	49.0	1.00
**Religion**				
Hindu^a^	8,232	53.7	46.3	1.00
Muslim	2,113	44.9	55.1	0.76** [0.69, 0.84]
Christian and others	1,487	43.1	56.9	0.65*** [0.58, 0.73]
**Hospitalised in the past year**				
No	10,863	50.2	49.8	1
Yes	969	49.8	50.2	1.22*** [1.07, 1.40]
**Total**	11,832	50.2	49.8	

Excluded non-PFHIS(*n* = 1222)

*AOR* Adjusted Odds Ratio, *95%CI* 95% Confidence Interval

*p < 0.05

**p < 0.01

***p < 0.001

^a^Reference category

### Inequalities in PFHIS coverage

**Fig. 1:**
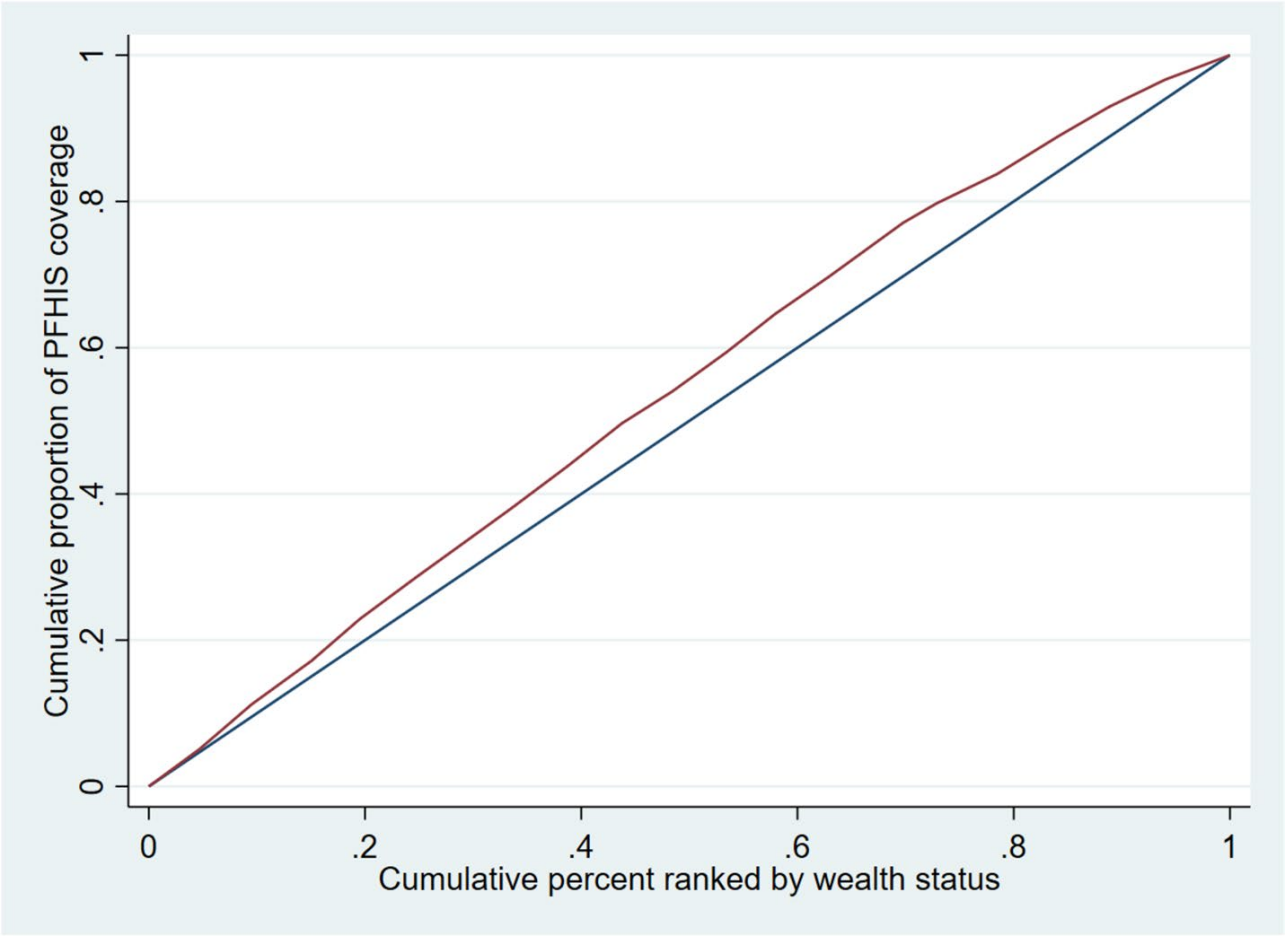
Concentration curve for PFHIS coverage in Kerala

**Fig. 2:**
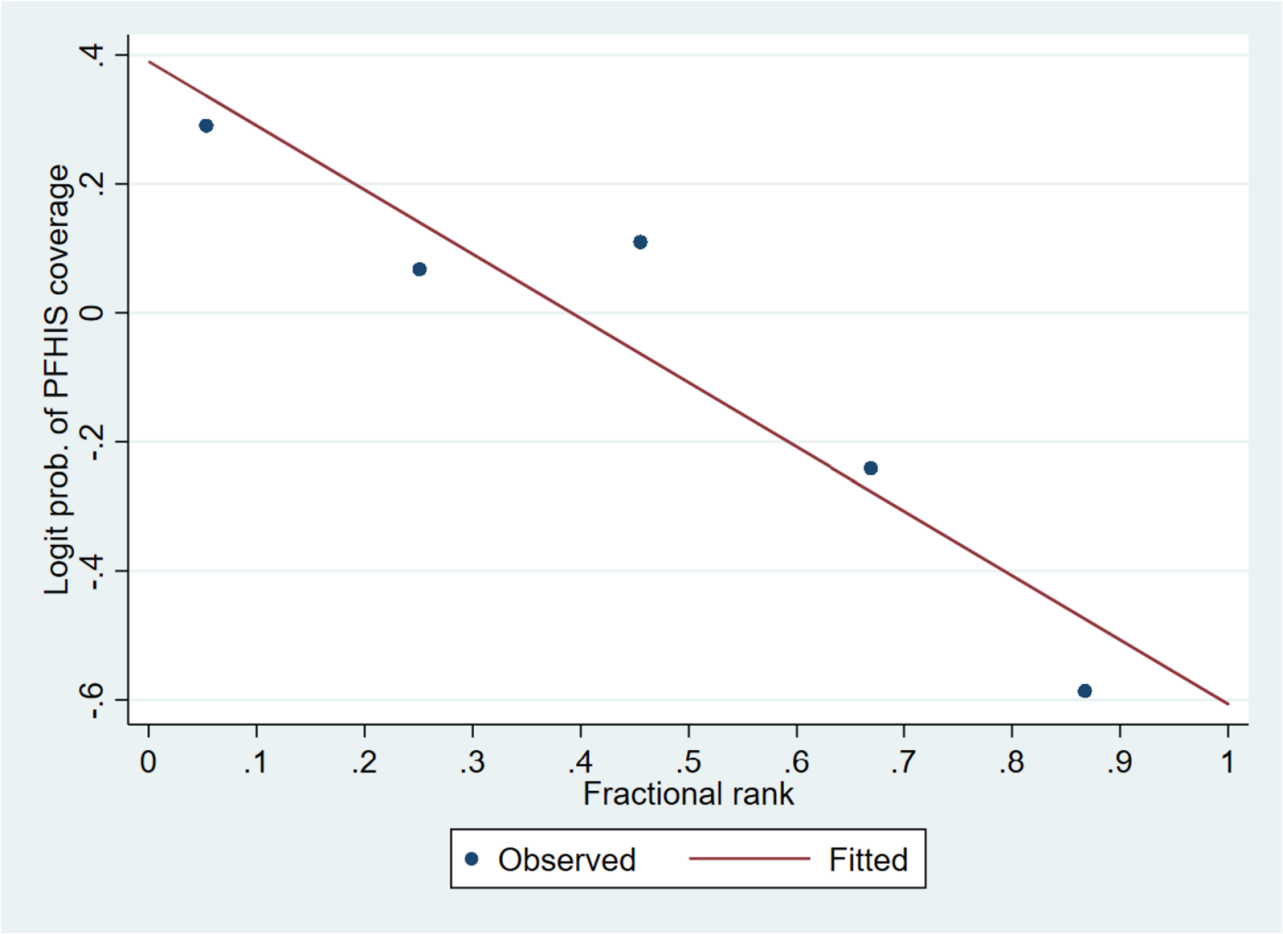
Slope index of inequality for PFHIS coverage in Kerala

Table 3, Figs. 1 and 2 shows the absolute (SII), and relative (CIX) measures of economic inequality for PFHIS coverage by socio-demographic characteristics. Overall, negative magnitude of RCI [-16.8% (95%CI: -25.3, -8.4)] and SII [-21.5% (95%CI: -36.1, -7.0)] suggests a higher concentration of PFHIS coverage among the poor. Absolute and relative economic inequality in PFHIS coverage was higher in females [SII: -26.7% (95%CI: -37.8, -15.6); RCI: -18.2% (95%CI: -26.3, -10.1)] as compared to males [SII: -21.5% (95%CI: -36.1, -7.0); RCI: -15.2% (95%CI: -25.5, -4.9)]. Absolute and relative economic inequality in PFHIS coverage was significantly higher among those who were hospitalized in the past year [SII: -40.4% (95%CI: -63.9, -16.8); RCI: -28.8% (95%CI: -45.7, -12.1)] as compared to those who were not hospitalised. Among SC and ST sub-groups, economic inequality was not significant. Other (majority) social groups had higher absolute [SII: -29.4% (95%CI: -51.1, -7.7)] and relative economic inequality [RCI: -19.7% (95%CI: -34.5, -4.5)] in PFHIS coverage as compared to other groups. Among Muslims, absolute [SII: -35.1% (95%CI: -54.2, -16.0)] and relative economic inequality [RCI: -22.6% (95%CI: -35.7, -9.5)] was highest compared to Hindus and Other religious groups.

**Table 3:** Socioeconomic inequality in PFHIS coverage by background characteristics in Kerala

Background characteristics	RCI [95%CI]	SII [95%CI]
**Gender**		
Male	-15.2 [-25.5, -4.9]	-21.5 [-36.1, -7.0]
Female	-18.2 [-26.3, -10.1]	-26.7 [-37.8, -15.6]
**Marital Status**		
Never married	-18.0(-28.6, -7.5)	-25.1(-40.5, -9.6)
Currently Married	-16.0(-25.1, -6.9)	-23.1(-36.0, -10.2)
Currently not married	-17.8(-27.1, -8.4)	-26.1(-38.4, -13.7)
**Hospitalised in the past year**		
No	-15.7(-24.6, -6.8)	-22.7(-35.1, -10.4)
Yes	-28.8(-45.7, -12.0)	-40.4(-63.9, -16.8)
**Social Group**		
Scheduled Caste (SC)	-4.5(-31.2, 22.2)	-0.7(-40.5, 39.2)
Scheduled Tribe (ST)	18.8(-12.7, 50.2)	18.2(-42.9, 79.4)
Other Backward Class (OBC)	-18.0(-25.7, -10.3)	-26.3(-37.0, -15.6)
Others	-19.7(-34.9, -4.5)	-29.4(-51.1, -7.7)
**Religion**		
Hindu	-15.0(-24.7, -5.3)	-21.3(-34.0, -8.5)
Muslim	-22.6(-35.7, -9.5)	-35.1(-54.2, -16.0)
Christian and others	-12.1(-28.4, 4.1)	-14.4(-38.5, 9.7)
**Overall**	**-16.8 [-25.3, -8.4]**	**-24.3 [-36.1, -12.5]**

### Hospitalization by PFHIS coverage

Table 4 presents the distribution of respondents hospitalised in the past year by PFHIS coverage according to their socio-demographic characteristics. A total of 55.7% of respondents who were hospitalized were covered by PFHIS. Compared to females, males who were hospitalized in the past year had higher PFHIS coverage (59.7% vs. 52.4%). Respondents who were hospitalised in the past year from the poorest wealth quintile (68.0%) had the highest PFHIS coverage, followed by poorer (60.2%), middle (57.9%), richer (47.0%) and richest wealth quintile (41.1%). PFHIS coverage was highest among SC respondents hospitalised in the past year (64.7%), followed by OBC (55.2%). Hindu respondents (60.4%) had higher PFHIS coverage compared to Muslim (50.2%), Christian and other religious groups (44.1%) who were hospitalised in the past year. Notably, respondents hospitalised in public facilities (62.9%) had higher PFHIS coverage compared to respondents hospitalised in private facilities (48.3%).

**Table 4:** Percentage distribution of respondents hospitalized in the past year by PFHIS coverage in Kerala, 2019

**Background characteristics**	PFHIS coverage (%) [531]	No insurance (%) [420]	N
Gender			
Male	59.7	40.3	433
Female	52.4	47.6	518
Marital Status			
Never married	52.5	47.5	81
Currently Married	58.0	42.0	645
Currently not married	49.4	50.6	225
Wealth Quintile			
Poorest	68.0	32.0	217
Poorer	60.2	39.8	199
Middle	57.9	42.1	204
Richer	47.0	53.0	193
Richest	41.1	59.0	138
Social Group			
Scheduled Caste (SC)	64.7	35.3	62
Scheduled Tribe (ST)	52.8	47.2	18
Other Backward Class (OBC)	55.2	44.8	656
Others	54.8	45.2	215
Religion			
Hindu	60.4	39.6	656
Muslim	50.2	49.8	171
Christian and others	44.1	55.9	124
Health Facilities			
Private	48.3	51.8	476
Public	62.9	37.1	475
Total	55.7	44.3	951

### Out-of-pocket expenditure for hospitalization

Table 5 presents the mean and median OOPE for hospitalisation among those covered by PFHIS and not covered by any insurance. Overall, PFHIS was associated with lower OOPE across all groups. The median OOPE for hospitalisation at private health facilities was INR 9000 (∼USD 10.96) among those covered by PFHIS, whereas it was INR 10500 (∼USD 127.91) at private health facilities among those not covered by insurance. Among those who covered by PFHIS, median OOPE was higher among males (INR 3300, ∼ USD 40.20) compared to females (INR 1900, ∼USD 23.15), whereas median OOPE was slightly higher among females (INR 6000, ∼USD 73.09) as compared to males (INR 5750, ∼USD 70.04) who were not covered by any insurance. The median OOPE for hospitalisation was higher among married participants as compared to other categories of marital status, weather covered by PFHIS (INR 3000, ∼USD 36.54) or not covered by any insurance (INR 6000, ∼USD 73.09). Median OOPE for hospitalisation was zero among SC groups, whether covered by PFHIS or not covered by any insurance. Among those covered by PFHIS, the median OOPE for hospitalisation among OBC social group (INR 2900, ∼USD 35.33) was comparatively higher than the other social caste group. Similarly, among those covered by PFHIS, the median OOPE was highest among Muslims (INR 5000, ∼USD 60.91), whereas, for those not covered by insurance, the median OOPE was highest among Christians and other religious groups (INR 8000, ∼USD 97.45). Median OOPE for hospitalisation was highest among the richest wealth quintile whether covered by PFHIS (INR 4600, ∼USD 56.04) or not (INR 9000, ∼USD 109.63). The payment threshold of INR 4600 (USD 56.04) was crossed by other wealth quintiles not covered by any insurance (i.e., richer (INR 6000, ∼USD 73.09) and poorer wealth quintiles (INR 5300, ∼USD 64.56)).

**Table 5:** Mean and median out-of-pocket expenditure for hospitalization in PFHIS vs. non-PFHIS/no insurance coverage

Characteristic	Covered by PFHIS			No insurance		
	**N**	**Median OOPE (INR)**	**Mean**	N	**Median OOPE (INR)**	**Mean**
Health facilities						
Private	231	9000	25942	245	10500	23338
Public	300	0	4170	175	450	7167
Gender						
Male	255	3300	15654	178	5750	21480
Female	276	1900	11576	242	6000	13137
Marital Status						
Never married	45	1500	9766	36	5000	37565
Currently Married	377	3000	15369	268	6000	16668
Currently not married	109	2000	8308	116	3450	8840
Social Group						
Scheduled Caste (SC)	43	2000	9658	19	900	3920
Scheduled Tribe (ST)	9	0	2152	9	0	4861
Other Backward Class (OBC)	363	2900	12043	293	6000	17231
Others	116	2750	20226	99	7700	17874
Religion						
Hindu	390	2500	13889	266	4000	16553
Muslim	84	5000	13753	87	7300	17120
Christian and others	57	2000	11362	67	8000	15811
Wealth Quintile						
Poorest	143	3000	9930	74	2350	10426
Poorer	117	0	6103	82	5300	23794
Middle	118	4000	14291	86	3400	8452
Richer	97	2700	19965	96	6000	17927
Richest	56	4600	25376	82	9000	20260
Overall	531	2880	13534	420	5800	16527

## Discussion

PFHIS was intended to play an integral role in moving towards UHC. Our study found a substantial proportion of respondents covered under PFHIS in the state. This is consistent with the findings from the latest National Family Health Survey (NFHS-5) which reports more than 50% of households (51.5%) in the state with at least one member covered by health insurance [49]. A higher proportion of participants in the lowest quintiles (poorest, poor & middle) were found to be covered under PFHIS in the state, which is a feature of the design of the insurance and is to be expected. Across social groups, SC and OBC households had higher coverage, yet insurance coverage was found to be relatively lower among the ST households in the state. A study examining the impact of RSBY/CHIS scheme in the state reported that the most marginalized population in the state like the SC, households are left behind from getting enrolled under the scheme [50]. A study conducted by Neena et al. 2015, in Kerala reported similar findings [51]. This said, median costs incurred in this group with or without insurance was zero, suggesting mechanisms for financial protection likely exist for them independent of insurance (we discuss this more later).

NITI Aayog, the official “think tank” of Government of India, in their 2021 report on health insurance for India’s missing middle concluded that at least 30% of the Indian population was not covered under any health insurance, which is spreads across all income quintiles in urban and rural areas [52]. Results from a 2021 study by Singh and colleagues in India showed that states with higher penetration of PFHIS among richer quintiles have failed to cover disadvantaged populations [53]. Similar results have been seen in studies in LMICs: PFHIS enrolment often misses disadvantaged populations and is instead covering wealthier quintiles [54, 55]. Studies cite lack of awareness and political will as major reasons for low coverage of eligible populations [56, 57].

This is a dynamic area of policymaking as well. To universalise coverage, in 2020, the state of Kerala decided to converge all the Government sponsored health care schemes like Comprehensive Health Insurance Scheme-CHIS, Senior Citizen Health Insurance Scheme-SCHIS, Karunya Benevolent Fund-KBF. Ayushman Bharat Pradhan Mantri Jan Arogya Yojana (PMJAY) and Karunya Arogya Suraksha Padhathi (KASP) [58, 59]. However, the beneficiary base for this converged scheme remains the same as for individual sub-schemes in 2023, meaning that eligible populations who were already left behind do not yet have an option to enrol and be covered. Apart from being an obvious area of policy making and/or adaptation, further study by way of evaluation or time-series analysis may shed light on how scheme convergence affects health seeking and health of populations (or not, as the case may be). This may lead to further insights on how both breadth and depth of coverage may be optimized. Moreover, research should explore community preferences and motivations for not utilising PFHIS, even if enrolled.

We found a considerable difference in hospitalization between males and females covered by PFHIS. A higher proportion of males sought care through PFHIS even when our study showed higher coverage of PFHIS among females in the state. The higher coverage of females under PFHIS is consistent with the findings of studies from LMICs [60–62]. The utilization pattern of PFHIS among females in Kerala needs to be further explored. Findings from a study in Tamil Nadu, nearby south Indian state showed higher enrolment coverage among females and significantly lesser utilization than males [63]. Another study in Tamil Nadu by Ramakrishnan and colleagues concluded that gender barriers at the household, community and programme level were associated with reduced uptake of hospitalisation among PHFIS females enrollees [64]. Further mixed methods research can shed light on whether/how barriers are shared or unique across these two southern states.

Our study also found that found that hospitalization through PFHIS was higher among populations belonging to the poorest quintile followed by poorer and middle. This is a positive sign suggesting that there is access to insurance among disadvantaged populations in the state. Studies from India and other countries have found that health insurance has increased the portability of seeking care and reduce delays [65–69]. Private sector facilities were found to be the most preferred among both insured and non-insured populations due to ease of access and perceived quality of care in Kerala [70]. Adding to this, since insured populations actually have the choice to choose between public and empanelled private facilities, [69] insured persons from the lowest socio-economic strata may be exercising this choice and choosing private sector facilities in greater numbers, as has been seen in other LMICs as well [71–74]. While analysing the share of hospitalization among public and private sector facilities, we found that participants hospitalized in public hospitals had higher chance of getting benefits through PFHIS (62.9% vs 48.3%). This can be attributed to the availability, access and equitable distribution of public and private empanelled healthcare facilities having specialities for which the population seek care. A study by Joseph and colleagues, found that more than half of the empanelled facilities under ABPMJAY were public sector facilities and only 14% of them offered care for all specialities covered under the scheme [75]. A study done in Chhattisgarh reported low distribution of empanelled private hospitals in disadvantaged districts,^6^ where eligible population numbers were, as compared to public sector hospitals, which were evenly distributed in disadvantaged districts [76]. Equitable insurance coverage does not always ensure equitable access to healthcare, while it is dependent on the availability of service providers which needs to be taken care for achieving UHC. Greater analysis of foregone care using population-based studies could help shed light on this.

In our study we found that median OOPE for hospitalization among insured participants was marginally lower than those not covered by insurance for both sexes. However, higher median OOPE was observed among insured males, whereas uninsured females had higher slightly spending for hospitalization as compared to uninsured males. This is consistent with the findings of National Health Accounts 2018–19 which reports people in Kerala were reportedly spending 2.5 times more than those in other southern states of India [77]. Studies in LMICs have reported despite being covered by PFHIS, patients incur OOPE [55, 67, 68, 78–81]. This may be attributed to package design (which is focused on inpatient care only and even in that context, may not cover the entire gamut of expenses incurred) – this is another area that requires study using mixed methods. We also found zero OOPE reported by SC groups irrespective of their coverage under PFHIS. Apart from being covered under PFHIS, this may be attributed to the healthcare scheme implemented by the Scheduled Tribes Development Department which provides free diagnosis, treatment, medication, medical aids, transportation, food expenses and pocket money for bystanders during hospitalization [82, 83].

To summarize, PFHIS are tailormade to cover economically and socially disadvantaged sections of the society: it is therefore important to estimate their actual reach and coverage among eligible populations. This requires research to estimate the true denominators of eligible populations; one such exercise has been underway through the Kerala “poorest households” initiative, which may serve as a proxy for many (but not all) groups facing disadvantage [84]. Also, it is important to understand the experiences and pathways of catastrophic health expenditure, through which population which are pushed to impoverishment due to health expenditure and ensure these households are covered through PFHIS. It is also important to continue to monitor the availability and distribution of empanelled health care providers under PFHIS in the state, as well as the services coverage they offer, which are vital in ensuring access as well as financial risk protection.

## Limitations

Our study revealed several important findings about the status of implementation of PFHIS in Kerala. However, there are some limitations that warrant mention. Our study measured self-reported coverage and expenses, which can lead to over- or underestimation because of recall bias. We did not gather or compute data on place of residence and thus did not examine rural–urban differences in health insurance coverage in the four districts: this could be explored further and will likely require differentiated sampling for urban and rural areas. As aforementioned, we were underpowered to look at differences in coverage, utilisation, and spending among trans persons in our sample. Future research should explore these population subgroups specifically to arrive at key drivers of under enrolment, under-utilisation, and expenditure.

## Conclusion

Despite most of the participants being enrolled under PFHIS insurance in Kerala, our study found that OOP expenses continue to burden households across four districts of the state. While PFHIS seems to be appropriately targeting poorer populations, among the insured, the greatest costs are borne by populations with historical advantage. Among the uninsured, wealthier population subgroups were spending the greatest amount, raising questions about whether those in poorer income groups were forgoing care altogether or seeking care using cheaper, public avenues.

## Data Availability

All datasets used for supporting the conclusions of this paper are available from the corresponding author on request.

## Abbreviations

SDGs: Sustainable Development Goals
UHC: Universal Health Coverage
PFHIS: Publicly Funded Health Insurance Schemes
RSBY: Rashtriya Swasthya Bhima Yojana
OOPE: Out-of-Pocket Expenditure
BPL: Below Poverty Line
CHIS: Comprehensive Health Insurance Scheme
APL: Above Poverty Line
ESIS: Employees State Insurance Scheme
CGHS: Central Government Health Scheme
NHA: National Health Accounts
NSSO: National Sample Survey Organization
FHC: Family Health Centre
PHC: Primary Health Centre
PMJAY: Pradhan Mantri Jan Arogya Yojana
KASP: Karunya Arogya Suraksha Paddathi
MEDISEP: Medical Insurance for State Employees and Pensioners
SC: Scheduled Caste
ST: Scheduled Tribe
OBC: Other Backward Class

## Footnotes

1 Individuals or households whose consumption expenditures fall below the poverty line of $1.90 per person per day are classified as "Below the Poverty Line (BPL)" and considered to be living in poverty.

2 On a family floater basis, the entire benefit package can be utilized by either one or all members of the family.

3 The state introduced another financial risk protection mechanism soon after this expansion, namely the Karunya Benevolent Fund (KBF). The KBF initiative was put in place by the Department of Lotteries and is funded by profits from the sale of Karunya weekly lotteries. It offers a one-time financial aid of 2 lakhs to families with an annual income below 3 lakhs who are dealing with high-cost health conditions [30, 31].

4 We asked each participant to self report their gender. We had a sample of 10 persons who identified as trans. Given this relatively small sample size and our analytical approach, we regrettably had to remove them from analysis.

5 Scheduled Tribe’ and ‘Scheduled Caste’ are the tribal and caste groups recognized by the President of India according to article numbers 341 and 342 of the Constitution of India [40]. ‘Backward Class’ is the term used by the Government of India to classify groups that are educationally or socially disadvantaged [41].

6 The vulnerability tertiles of the districts were determined by mapping the availability of public and private facilities per 100,000 enrolled population.
